# Enhanced Visible Light Photocatalytic Activity of ZnO Nanowires Doped with Mn^2+^ and Co^2+^ Ions

**DOI:** 10.3390/nano7010020

**Published:** 2017-01-19

**Authors:** Wei Li, Guojing Wang, Chienhua Chen, Jiecui Liao, Zhengcao Li

**Affiliations:** 1 State Key Lab of New Ceramic and Fine Processing, School of Materials Science & Engineering, Tsinghua University, Beijing 100084, China; liwei941029@foxmail.com (W.L.); gj14@mails.tsinghua.edu.cn (G.W.); caramel6517@gmail.com (C.C.); liaojc14@mails.tsinghua.edu.cn (J.L.); 2 Key Lab of Advanced Materials Laboratory (MOE), School of Materials Science and Engineering, Tsinghua University, Beijing 100084, China

**Keywords:** ZnO, Mn^2+^ and Co^2+^ doping, photocatalytic activity

## Abstract

In this research, ZnO nanowires doped with Mn^2+^ and Co^2+^ ions were synthesized through a facile and inexpensive hydrothermal approach, in which Mn^2+^ and Co^2+^ ions successfully substituted Zn^2+^ in the ZnO crystal lattice without changing the morphology and crystalline structure of ZnO. The atomic percentages of Mn and Co were 6.29% and 1.68%, respectively, in the doped ZnO nanowires. The photocatalytic results showed that Mn-doped and Co-doped ZnO nanowires both exhibited higher photocatalytic activities than undoped ZnO nanowires. Among the doped ZnO nanowires, Co-doped ZnO, which owns a twice active visible-light photocatalytic performance compared to pure ZnO, is considered a more efficient photocatalyst material. The enhancement of its photocatalytic performance originates from the doped metal ions, which enhance the light absorption ability and inhibit the recombination of photo-generated electron-hole pairs as well. The effect of the doped ion types on the morphology, crystal lattice and other properties of ZnO was also investigated.

## 1. Introduction

While enjoying the rapid development of human society, people are also suffering from the accompanying environmental problems, for instance air pollution, water pollution and so on. As a result, developing a pollution-free and environmentally friendly technology for environmental cleanup and supplying sustainable and clean energy have become emergent tasks in the past few years. In 1972, Fujishima et al. found that TiO_2_ could produce hydrogen and oxygen through a photocatalytic reaction [1]. Since then, a series of semiconductor materials had been investigated for its application in the photocatalysis field [2,3,4,5,6]. These semiconductors can generate electrons and holes, which are highly active and able to degrade the pollutants in water with light illumination. Photocatalysis has quite a few advantages, including low cost, low toxic properties, low energy consumption, as well as no secondary pollution and reusability. In this regard, photocatalytic oxidation technology has been getting more and more attention.

Among all kinds of photocatalysts, ZnO, an n-type semiconductor with a wide direct bandgap (about 3.37 eV), is widely used for degrading pollutants in water. Owing to its non-toxic properties, long-term stability, high carrier mobility, low cost and biocompatibility [7], ZnO also has many applications in other areas, such as gas sensors [8], solar cells [9], H_2_ studies [10], field-effect transistors [11], ultraviolet lasers [12] and light-emitting diodes [13]. However, some drawbacks of ZnO limit its utilization in photocatalysis. Due to its wide bandgap, ZnO can only be activated in the presence of UV light, which constitutes only about 5% of the solar light energy reaching Earth’s surface. Moreover, the photo-excited electron-hole pairs recombine fast on the surface of ZnO, limiting its photocatalytic properties.

In order to cope with these problems, doping with transition metals is employed to improve the optical properties of ZnO and to inhibit the recombination of photo-generated charge carriers. Its enhancement in the visible light photocatalytic performance can be ascribed to the metal ions substituted in the lattices of ZnO, which narrow the bandgap of ZnO and increase the delocalization of electrons in the nanostructure. Enhanced photocatalytic performances have been observed previously for ZnO doped with Fe [14], Ni [15], Cu [16], Cr [17] and Mg [18] ions. For instance, Saleh R. et al. synthesized Fe-doped ZnO nanoparticles with a co-precipitation method and researched the effects of pH, dopant concentrations and photocatalytic dosage [14]. Mohan R. et al. synthesized Cu-doped ZnO nanorods through the vapor transport method and evaluated their photocatalytic performances with different Cu concentrations [16]. Wu C. et al. fabricated Cr-doped ZnO nanowires with high photocatalytic activity by a solvothermal route [17]. Among the transition metals, the ionic radius and covalent radius of Co^2+^ (0.72 Å and 1.16 Å) and Mn^2+^ (0.80 Å and 1.17 Å) are close to those of Zn^2+^ (0.74 Å and 1.25 Å). Hence, it is easy for Co^2+^ and Mn^2+^ ions to replace Zn^2+^ ions in the ZnO lattice without causing much lattice deformation. Moreover, due to the Co 3d ligand field split states [19], some absorption peaks occur in the visible light range, enhancing its visible light absorption ability. Therefore, Co and Mn are considerable candidates for doping into the ZnO matrix.

The photocatalytic performance with the visible light illumination of Mn- or Co-doped ZnO nanoparticles [20,21], nanoflowers [22], nanodisks [23], nanorods [23,24] and nanoplates [25] has been reported previously. However, there are few studies on the visible light photocatalytic activity of Mn- or Co-doped ZnO nanowires grown on substrates. Compared to other nanostructures, applying ZnO nanowires on substrates for photocatalysis has advantages such as convenience for recycling and good reusability. Furthermore, the photocatalytic activity of ZnO is closely related with its size and morphology [26,27,28], making it reasonable to study the performance of ZnO-based photocatalysts with different aspect ratios when compared to the previous reports. As the Mn^2+^ has a larger ionic radius than Zn^2+^, while the ionic radius of Co^2+^ is smaller than that of Zn^2+^, the difference of their effect on ZnO nanowires is also considered.

Based on the aforementioned points, Mn-doped and Co-doped ZnO nanowires were synthesized via facile and low-cost hydrothermal methods in this work. Their photocatalytic performances were investigated by conducting methyl orange (MO) degradation with visible light irradiation. By comparing the morphology, crystal lattice and other properties of Mn-doped and Co-doped nanowires, the effect of the type of doping ions was studied as well.

## 2. Materials and Methods

### 2.1. Materials

The zinc nitrate hexahydrate (Zn(NO_3_)_2_·6H_2_O, Xilong Chemical Co., Ltd., Beijing, China), manganese nitrate hexahydrate (Mn(NO_3_)_2_·6H_2_O, Beijing Modern Oriental Fine Chemistry Co., Ltd., Beijing, China), cobalt nitrate hexahydrate (Co(NO_3_)_2_·6H_2_O, Beijing Modern Oriental Fine Chemistry Co., Ltd., Beijing, China), Zinc oxide ceramic target (Chino Advanced Materials Beijing technology Co., Ltd., Beijing, China), acetone (CH_3_COCH_3_, Beijing Chemical Works, Beijing, China), ethanol (C_2_H_5_OH, Beijing Chemical Works, Beijing, China), concentrated ammonia (NH_4_OH, Beijing Modern Oriental Fine Chemistry Co., Ltd., Beijing, China), methyl orange (C_14_H_14_N_3_SO_3_Na, AR, Beijing Modern Oriental Fine Chemistry Co., Ltd., Beijing, China). All reagents were analytical reagent and used as received without any further purification.

### 2.2. Preparation Ion of ZnO Seed Layer

A seed layer of ZnO was deposited on the substrate in order to help the nanowires grow better. First, the substrates were successively cleaned by acetone, ethanol and de-ionized water for 15 min, respectively; second, the ZnO seed layer was uniformly deposited on the cleaned substrates through radio frequency (RF) magnetron sputtering for 7 min. The other parameters were as follows: The system pressure, 2.5 × 10^−4^ Pa; RF power, 80 W; O_2_ flow rate, 10 sccm; Ar flow rate, 30 sccm; and working pressure, 0.6 Pa.

### 2.3. Synthesis of Mn^2+^- and Co^2+^-Doped ZnO Nanowires

Doped and undoped ZnO nanowires were grown on the Si substrates by a seed-assisted hydrothermal method. A typical synthesis process is briefly described as following. First, 80 mL of aqueous precursor solution (0.075 M) was prepared by mixing zinc nitrate hexahydrate and manganese (or cobalt) nitrate hexahydrate at the molar ratio of 9:1 in deionized water. Then, ammonia was added to the solution drop by drop to adjust the pH value to 10. The solution was stirred at room temperature for half an hour until its color turned brown. Finally, the as prepared substrates were vertically inserted into the solution in a Teflon-lined autocave and heated at 90 °C for 6 h. The prepared samples were rinsed with distilled water and dried at room temperature. Undoped ZnO nanowires were prepared by the same method without adding manganese (or cobalt) nitrate hexahydrate in the precursor solution.

### 2.4. Characterization

The crystal structures of the samples were characterized by X-ray diffraction (XRD, Rigaku Smart Lab, Tokyo, Japan) employing Cu Kα radiation at a scanning rate of 0.1 s^−1^ in the 2θ range of 20°–60°. The morphology of the ZnO nanowires was examined by scanning electron microscopy (SEM, JEOL-JSM 7001F, Tokyo, Japan). The elemental composition was evaluated by energy dispersive X-ray spectroscopy (EDS) (JEOL, Tokyo, Japan). The chemical states on the surface were analyzed by X-ray photoelectron spectroscopy (XPS, ESCALAB250Xi, Thermo, Waltham, MA, America), using the signal of C 1s (284.8 eV) as reference to calibrate the binding energy. Photoluminescence (PL) spectrum was obtained using a Lab Ram HR Evolution Micro Raman Spectrometer. UV-Vis absorption spectrum and the degradation rate of methyl orange (MO) were measured by a UV-Vis spectrophotometer (UV-2600, SHIMADZU, Tokyo, Japan) in the range of 300–800 nm.

### 2.5. Photocatalytic Performance

The standard method for the photo-degradation of MO was used to determine the photocatalytic activity. For testing photocatalytic performance, the ZnO nanowires were grown on Si substrates with the size of 1.5 × 1.5 cm^2^. Before the test, each sample was immersed into 5 mL methyl orange solution (10 μmol·L^−1^) in darkroom for 1 h. After acquiring an adsorption and desorption equilibrium, each sample was removed to another 5 mL methyl orange solution. A 50 W Xe light equipped with a UV cut-off filter (λ > 400 nm) was used for illumination. The test continued for 75 min and the absorption spectrum of the MO solution was measured each 15 min. According to the Lambert–Beer law, the photocatalytic activity can be obtained from the absorption spectrum [29]. The degradation rate of MO was calculated using Equation (1):

*D* = (*c*_0_ − *c_t_*)/*c_0_*~(*A*_0_ − *A_t_*)/*A*_0_ (1)

where *c*_0_ and *A*_0_ are the concentration and absorption of MO before illumination, while *c_t_* and *A_t_* are the concentration and absorption of MO after illumination.

## 3. Results and Discussion

### 3.1. Characterization

**Figure 1:**
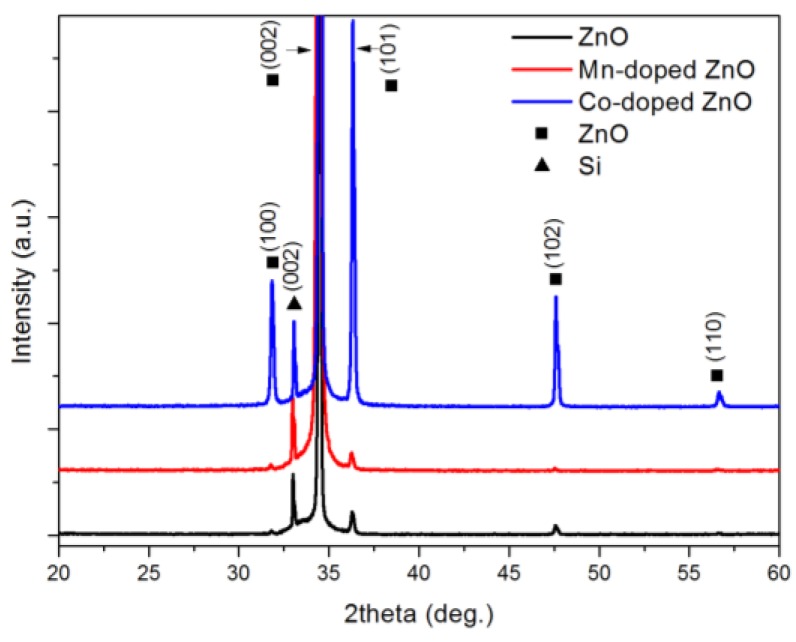
X-ray diffraction (XRD) pattern of ZnO, Mn-doped ZnO and Co-doped ZnO.

The XRD patterns of ZnO nanowires, Mn-doped ZnO nanowires and Co-doped ZnO nanowires are shown in Figure 1. The diffraction peaks of the ZnO samples could be indexed to the hexagonal wurtzite structure of ZnO (JCPDS 36-1451). The XRD patterns of Mn-doped and Co-doped ZnO nanowires had the same diffraction peaks as ZnO. There were no other peaks to be attributed to manganese or cobalt compounds in the XRD patterns, indicating that doped Mn^2+^ and Co^2+^ ions did not change the crystalline structure of ZnO. All the diffraction peaks were sharp, revealing that all the samples have high crystallinity. Meanwhile, the relatively high intensity of the (002) peak of all the samples above implied a preferred orientation and anisotropic growth of all the ZnO samples. Furthermore, the (002) peak of Mn-doped ZnO nanowires moved slightly toward the side with a lower 2θ value, indicating the increment of lattice constants of Mn-doped nanowires. It comes from the fact that the Mn^2+^, which replaces the position of Zn^2+^ in the ZnO lattice, has a larger ionic radius. A similar peak shift was also observed in the XRD pattern of Co-doped ZnO nanowires, but the (002) peak was shifted in the opposite direction owing to the ionic radius of Co^2+^ being smaller than that of Zn^2+^. The significant increase of the (100), (101), (102) and (110) peaks in the Co-doped ZnO showed that the radial growth of ZnO was promoted by Co^2+^. Meanwhile, the slight decrease of these peaks in Mn-doped ZnO nanowires indicated that Mn^2+^ can inhibit the radial growth of ZnO. Their effect on the radial growth can also be seen from the diameter change of the ZnO nanowires mentioned afterwards.

**Figure 2:**
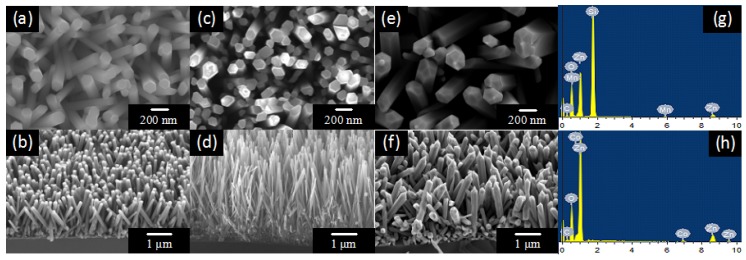
Scanning electron microscopy (SEM) images of the top and side view of (**a**,**b**) ZnO; (**c**,**d**) Mn-doped ZnO; (**d**,**e**) Co-doped ZnO. Energy dispersive X-ray spectroscopy (EDS) pattern of (**g**) Mn-doped ZnO; (**h**) Co-doped ZnO.

The morphologies of pure ZnO nanowires, Mn-doped ZnO nanowires and Co-doped ZnO nanowires are shown in Figure 2. Figure 2a,b exhibits the top and cross-sectional views of the pure ZnO nanowires. It can be clearly seen that the ZnO nanowires grew uniformly on the Si substrates, with diameters of around 100 nm and lengths around 1.5 μm. ZnO nanowires have the shape of hexagonal prisms. The top and cross-sectional views of Mn-doped and Co-doped ZnO nanowires are displayed in Figure 2c–f. The morphology of doped ZnO nanowires remained as hexagonal prisms, showing that doping did not change the morphology of the ZnO nanowires. However, the diameters of Mn-doped ZnO nanowires decreased to around 80 nm and the diameters of Co-doped ZnO nanowires increased to about 200 nm. The EDS patterns of doped ZnO are shown in Figure 2g,h, and Zn, O, Mn and Co elements can be observed in the pattern. It proves that Mn^2+^ and Co^2+^ ions were successfully doped into ZnO nanowires. Table 1 depicts the atomic percentage of each element, indicating that the incorporation of Mn^2+^ is higher than that of Co^2+^.

**Figure 3:**
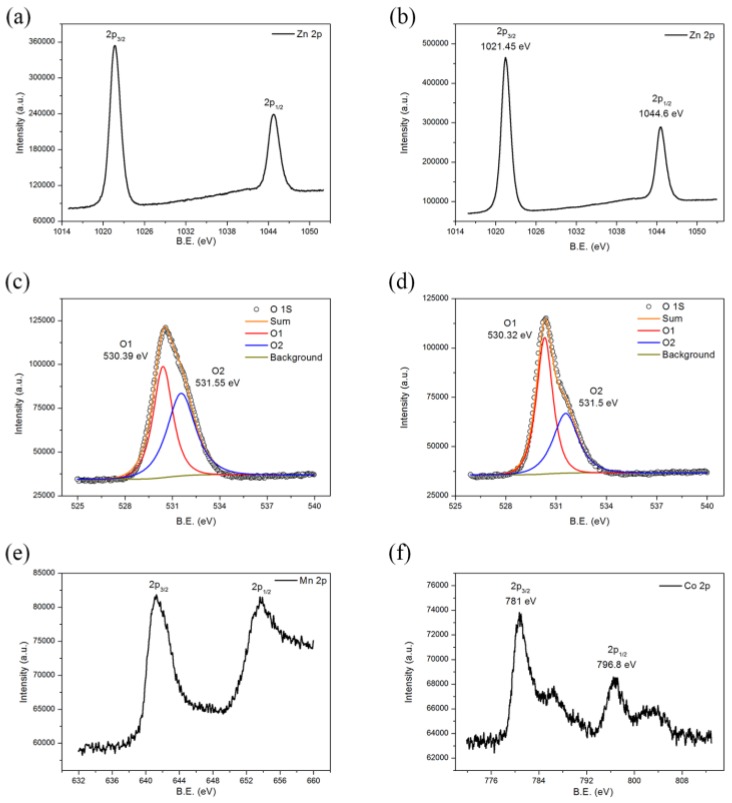
X-ray photoelectron (XPS) spectra of Mn-doped ZnO (**a**) Zn 2p; (**c**) O 1s; (**e**) Mn 2p and Co-doped ZnO (**b**) Zn 2p; (**d**) O 1 s; (**f**) Co 2p.

XPS was used to further prove the existence of Mn^2+^ and Co^2+^ ions in the ZnO crystal lattice and to detect the surface properties of doped ZnO nanowires. The XPS spectra of Mn-doped and Co-doped ZnO nanowires are shown in Figure 3. The Zn 2p_3/2_ and 2p_1/2_ signals appearing at ca. 1021 and ca. 1044 eV in doped ZnO can be attributed to Zn-O bonds in the ZnO lattice (Figure 3a,d) [30]. The high resolution signals of the O 1s orbital of Mn-doped and Co-doped ZnO are shown in Figure 3b,e, both of which can be divided into two peaks. The high intensity peak with a lower binding energy was due to the O^2−^ in the ZnO crystal lattice, while the broad peak resulted from the oxygen adsorbed on the surface of the ZnO nanowires [31,32]. The Mn 2p core level and the Co 2p core level are presented in Figure 3c,f. The signal-to-noise ratio of their XPS spectra was low because the amount of Mn^2+^ and Co^2+^ dopant ions was limited. The main peaks observed at 641.27 and 653.87 eV in the Mn 2p spectra corresponded to the binding energies of Mn 2p_3/2_ and 2p_5/2_, while the two peaks at 781.0 and 796.8 eV in the Co 2p spectra were associated with the binding energies of Co 2p_3/2_ and 2p_5/2_. The binding energies and energy separation (12.6 eV and 15.8 eV) of these peaks are characteristics of Mn^2+^ and Co^2+^, confirming that Mn^2+^ and Co^2+^ ions were successfully incorporated into the ZnO nanowires [32]. Besides, the presence of the shake-up satellite peaks in the Co 2p spectra also confirmed that the valence state of the Co element in doped ZnO nanowires was +2 as Co^3+^ would not have satellite peaks.

**Figure 4:**
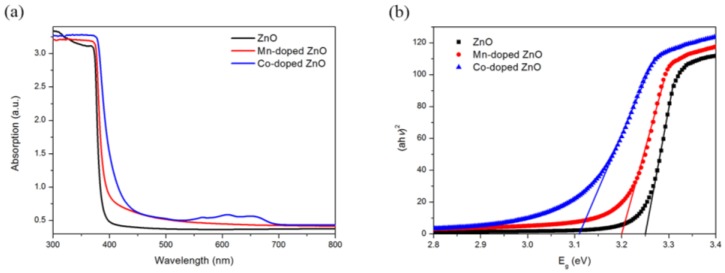
(**a**) UV-Vis spectra of ZnO, Mn-doped ZnO and Co-doped ZnO; (**b**) bandgap calculation figure.

Figure 4a exhibits the UV-Vis absorption spectrum of pure ZnO nanowires, and Mn-doped and Co-doped ZnO nanowires. The absorption edge of pure ZnO sample was 398 nm, which is consistent with its wide bandgap and reveals that its absorption was limited to the ultraviolet region. Compared to pure samples, doped ZnO nanowires both showed enhancement in the absorption in the UV-Vis region, while the absorption enhancement of Co-doped ZnO was more significant. Moreover, three absorption peaks occurred in the UV-Vis absorption spectra of Co-doped ZnO which revealed the Co 3d ligand field split states and confirmed the existence of Co^2+^ ions [27]. The bandgap of all the samples can be calculated according to Equation (2):

α = *K*(*hv* − *E_g_*)^1/2^/*hv* (2)

where α is the absorption coefficient, *K* is a constant, *hv* is the photon energy, and *E_g_* is the bandgap [33].

The *E_g_* of the samples can be acquired from the plot of (*ahv*)^2^ versus *hv* (Figure 4b). As shown in Figure 4b, the bandgap of Mn-doped and Co-doped ZnO nanowires decreased to 3.20 eV and 3.11 eV, respectively, which suggests that doping can narrow the optical bandgap of ZnO nanowires (3.25 eV). The decrease of the optical bandgap originated from the optically active sub-levels formed by doping. It is clear that the doping of Co^2+^ ions had a more remarkable influence on the optical properties than Mn^2+^ ions. Moreover, the colors of pure ZnO nanowires, Mn-doped ZnO nanowires and Co-doped nanowires were white, light yellow and green, respectively, presenting a tendency of becoming deeper. The darkening of the color also confirmed the enhancement in the visible absorption.

**Figure 5:**
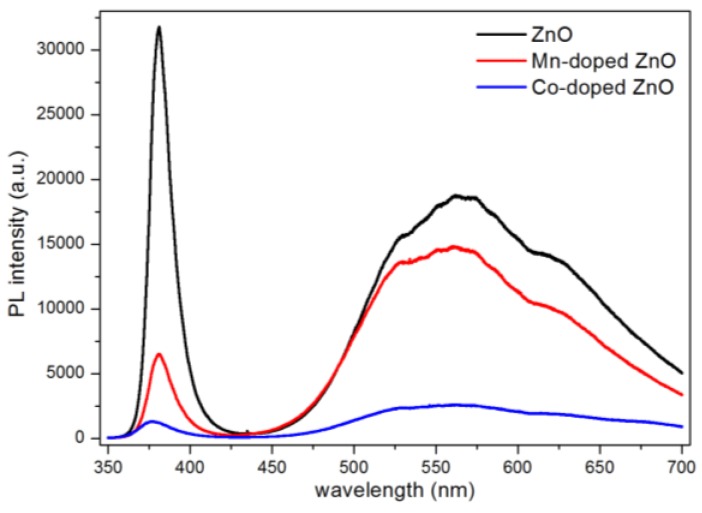
Photoluminescence (PL) spectrum of ZnO, Mn-doped ZnO and Co-doped ZnO.

A photoluminescence (PL) study at room temperature was used to investigate the optical properties of doped and undoped ZnO nanowires. The PL spectra are shown in Figure 5. Two peaks occurred in the PL spectra of pure ZnO nanowires. The peak of the strong emission locked at about 380 nm in the ultraviolet region originated from the recombination of free excitons in the near-band-edge of ZnO. The other peak which exhibited a broad emission at the green band was commonly attributed to the oxygen vacancies [34,35]. Photoluminescence is closely related with the recombination of the electron-hole pairs. It can be concluded that the weaker the PL intensity is, the slower the photo-generated electron-hole pairs recombine. The PL intensities of Mn-doped and Co-doped ZnO nanowires were both lower than that of pure ZnO nanowires, demonstrating that doped ZnO nanowires had an improvement in their optical properties. This is because that the doped ions provide several electron traps to suppress the recombination of electron-hole pairs. By comparing their PL intensities, it is obvious that Co-doped ZnO nanowires had a lower recombination rate and better optical properties.

### 3.2. Methyl Orange Photocatalytic Degradation

**Figure 6:**
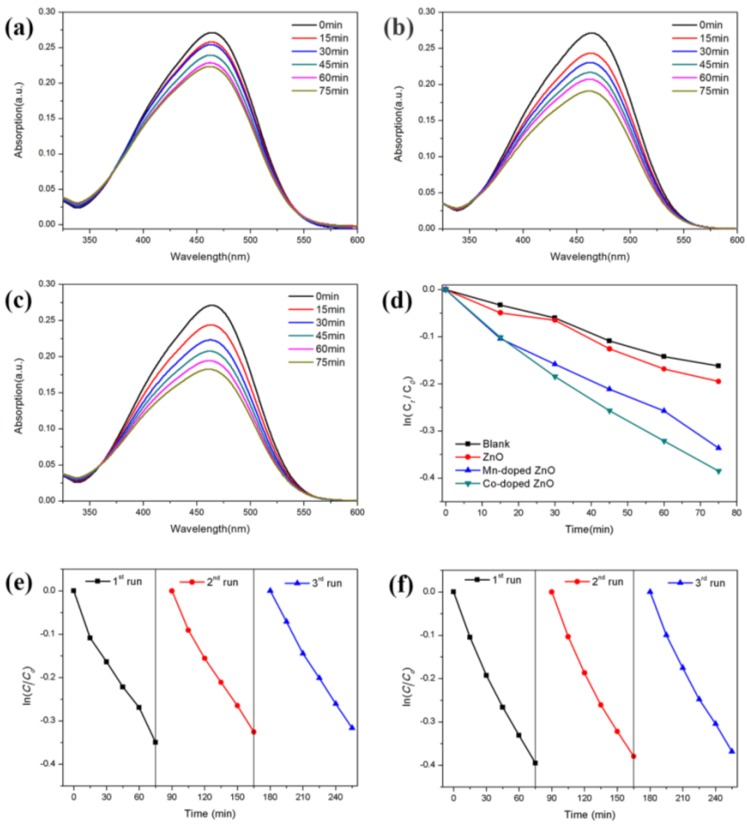
The absorption spectra of MO degraded by (**a**) ZnO; (**b**) Mn-doped ZnO; (**c**) Co-doped ZnO; (**d**) ln(*c_t_*/*c*_0_)-*t* figure. Cycling performance of (**e**) Mn-doped ZnO; (**f**) Co-doped ZnO.

The photocatalytic performances of all samples were calculated according to the Lambert-Beer law. Figure 6a–c displays the absorption spectra of MO degraded by pure ZnO nanowires, Mn-doped ZnO nanowires and Co-doped ZnO nanowires. As the relationship between ln(c*_t_*/*c*_0_) and time is nearly a linear relationship (Figure 6d), it can be concluded that the degradation reaction of MO corresponded to first-order reaction kinetics [4]. The first-order rate constants of pure ZnO nanowires, Mn-doped ZnO nanowires and Co-doped ZnO nanowires are shown in Table 2.

The pure ZnO nanowires have the worst photocatalytic activity because they can hardly absorb visible light. It can be clearly seen that the incorporation of Mn^2+^ and Co^2+^ into the ZnO crystal lattice effectively enhanced the visible light–driven photocatalytic activity of ZnO as both doped ZnO nanowires exhibited a better photocatalytic performance compared to bare ZnO nanowires. According to a previous report, commercial ZnO nanopowders can degrade only less than 10% MO in 90 min [36]. That is, the photocatalytic activity of doped ZnO nanowires is much higher than those of commercial ZnO nanopowders. Moreover, the rate constant of MO degradation for the Co-doped ZnO nanowires is nearly two times to that of pure ZnO nanowires, while the ratio of Mn-doped ZnO nanowires is 1.7. It shows that Co doping is more efficient in improving the visible light photocatalytic activity of ZnO nanowires than Mn doping. The better photocatalytic performance of Co-doped ZnO nanowires is due to its lower photo-excited electron-hole recombination rate and higher absorption in the visible light region, as shown in the results of the PL spectrum and UV-Vis spectrum before. Figure 6e,f shows the cycling performance of Mn-doped and Co-doped ZnO nanowires. The result of the photocatalytic degradation repetitive tests revealed that the photocatalytic performance of doped ZnO nanowires did not change much even after three runs, which means doped nanowires both had good cycling performances.

**Figure 7:**
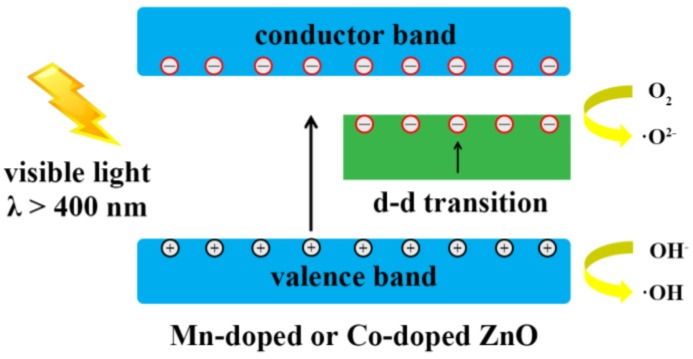
The photocatalytic mechanism of Mn-doped ZnO and Co-doped ZnO nanowires.

The mechanism of the photocatalysis of Mn-doped or Co-doped ZnO nanowires is exhibited in Figure 7. The doping of Mn^2+^ and Co^2+^ was able to modify the energy band structure of ZnO by introducing impurity energy levels within the bandgap of ZnO. Thus, the photo-generated electrons in the valence band of ZnO moved towards impurity energy levels or the conductor band and produced a large number of superoxide radicals (·O^2−^) under the irradiation of visible light, accompanied by the d-d transitions between the impurity energy levels. Meanwhile, the photo-excited holes were left in the ZnO valence band and moved to the surface of doped ZnO nanowires, forming highly oxidative hydroxyl radical species (·OH) after reacting with water molecules. The mechanism of the MO degradation can be described as follows:

ZnO + *hv* → e^−^ + h^+^ (3)

e^−^ + O_2_ → ZnO + ·O^2−^ (4)

h^+^ + H_2_O → H^+^ + ·OH (5)

h^+^ + OH^−^ → ·OH (6)

·OH + MO (Dye) → degradation products(7)

·O^2−^ + MO (Dye) → degradation products (8)

Hence, less energy was needed to excite electrons from the valence band, resulting in an improvement in the absorption ability of ZnO nanowires to visible light. That is, the optical bandgap was narrowed through Mn^2+^ or Co^2+^ ion doping. Besides, the doped Mn^2+^ or Co^2+^ ions can serve as electron traps. These trapping sites can restrain the recombination of photo-excited electron-hole pairs and promote the separation of photo-generated charge carriers. The two reasons mentioned above finally lead to an enhancement in the photocatalytic activity of doped ZnO nanowires under visible light.

## 4. Conclusions

In this study, ZnO nanowires were modified by Mn^2+^ and Co^2+^ doping via a convenient and cheap chemical method. Mn-doped and Co-doped ZnO nanowires both showed an enhancement in the visible light photocatalytic performance. Their superior photocatalytic activity can be ascribed to the dopant ions, which not only provide impurity energy levels to enhance the visible light absorption ability, but also serve as electron trapping sites to promote the separation and restrain the recombination of photo-generated charge carriers. Furthermore, Mn^2+^ and Co^2+^ doping have different influences on the ZnO nanowires in some respects. Mn^2+^ doping decreased the diameter of the nanowires and increased the lattice constants, while the effect of Co^2+^ doping was the opposite. According to the PL and UV-Vis absorption spectra, Co-doped ZnO has a lower photo-excited electron-hole pair recombination rate and higher visible light absorption. Therefore, the photocatalytic performance of ZnO had a greater enhancement after Co^2+^ doping than Mn^2+^ doping. Co-doped ZnO was twice as efficient in comparison to bare ZnO nanowires when serving as a photocatalyst, while the ratio of Mn-doped ZnO was 1.7. The result indicates that Co^2+^ is regarded as a better choice for metal ion doping for ZnO as a photocatalyst.

## Figures and Tables

**Table 1:** The atomic percentage of elements in Mn-doped and Co-doped ZnO nanowires.

Element	Atomic Percentage (%)	
	Mn-Doped ZnO	Co-Doped ZnO
C	9.31	13.50
Zn	43.23	40.97
O	44.73	44.84
Mn	2.72	/
Co	/	0.69

**Table 2:** The first-order rate constants of all the samples.

Sample	The First-Order Rate Constants (min^−1^)
ZnO	0.00265
Mn-doped ZnO	0.00436
Co-doped ZnO	0.00520
